# Endothelial cells stimulate growth of normal and cancerous breast epithelial cells in 3D culture

**DOI:** 10.1186/1756-0500-3-184

**Published:** 2010-07-07

**Authors:** Saevar Ingthorsson, Valgardur Sigurdsson, Agla JR Fridriksdottir, Jon G Jonasson, Jens Kjartansson, Magnus K Magnusson, Thorarinn Gudjonsson

**Affiliations:** 1 Stem cell research unit, Department of anatomy, Faculty of medicine, University of Iceland and Department of laboratory hematology, Landspitali, university hospital, (Vatnsmýrarvegur 16), Reykjavík, (101), Iceland; 2 Department of cellular and molecular medicine, Faculty of health science, Copenhagen University, (Blegdamsvej 3), Copenhagen, (2200), Denmark; 3 Department of pathology, Landspitali, university hospital, (building 7/8), Reykjavik, (101), Iceland; 4 St. Josefs hospital, (Suðurgata 41), Hafnarfjörður, (220), Iceland; 5 Department of pharmacology and toxicology, Faculty of medicine, University of Iceland, (Hofsvallagata 53), Reykjavík, (101), Iceland

## Abstract

**Background:**

Epithelial-stromal interaction provides regulatory signals that maintain correct histoarchitecture and homeostasis in the normal breast and facilitates tumor progression in breast cancer. However, research on the regulatory role of the endothelial component in the normal and malignant breast gland has largely been neglected. The aim of the study was to investigate the effects of endothelial cells on growth and differentiation of human breast epithelial cells in a three-dimensional (3D) co-culture assay.

**Methods:**

Breast luminal and myoepithelial cells and endothelial cells were isolated from reduction mammoplasties. Primary cells and established normal and malignant breast cell lines were embedded in reconstituted basement membrane in direct co-culture with endothelial cells and by separation of Transwell filters. Morphogenic and phenotypic profiles of co-cultures was evaluated by phase contrast microscopy, immunostaining and confocal microscopy.

**Results:**

In co-culture, endothelial cells stimulate proliferation of both luminal- and myoepithelial cells. Furthermore, endothelial cells induce a subpopulation of luminal epithelial cells to form large acini/ducts with a large and clear lumen. Endothelial cells also stimulate growth and cloning efficiency of normal and malignant breast epithelial cell lines. Transwell and gradient co-culture studies show that endothelial derived effects are mediated - at least partially - by soluble factors.

**Conclusion:**

Breast endothelial cells - beside their role in transporting nutrients and oxygen to tissues - are vital component of the epithelial microenvironment in the breast and provide proliferative signals to the normal and malignant breast epithelium. These growth promoting effects of endothelial cells should be taken into consideration in breast cancer biology.

## Background

The human breast gland is composed of two main cellular compartments, the branching epithelium, commonly referred to as the terminal duct lobular units (TDLUs) and the surrounding stroma. The TDLUs consist of an inner layer of luminal epithelial cells and an outer layer of myoepithelial cells separated from the surrounding vascular rich stroma by a basement membrane [1,2]. The breast stroma is composed of cellular components such as fibroblasts, immune cells and endothelial cells and the extracellular matrix (ECM) as well as entrapped growth factors within the ECM. Breast stroma accounts for roughly 80% of the total tissue volume and exerts a dominant effect on tissue morphogenesis in both the normal and malignant breast gland [3,4]. Recent studies have underscored the dominant role of breast stroma during epithelial morphogenesis (reviewed in [4]). Previous studies have shown that normal and malignant breast epithelium can mimic certain aspects of the breast gland histoarchitecture - such as lumen formation and branching morphogenesis - when cultured alone or in co-culture with fibroblasts in three-dimensional matrix [5-7]. The importance of the stroma in the normal and cancerous breast is becoming increasingly appreciated. Boulanger *et al. *demonstrated that spermatogonial cells underwent a breast epithelial differentiation program upon interaction with the mammary gland microenvironment [8]. Furthermore, Booth *et al. *showed that breast stroma can redirect neural progenitor cells to produce cellular progeny committed to breast epithelial differentiation [9]. While the functional role of fibroblasts and various extracellular matrix components in breast morphogenesis has been extensively studied [10-12], much less is known of the role of the vascular endothelium in the process. Previously, the role of endothelial cells has been seen as a passive conducting system, transporting oxygen and nutrients to tissues. In recent years however, studies in organogenesis and stem cell research have shown that endothelial cells play a pivotal role in tissue morphogenesis and stem cell niche [13,14]. In the prostate, vasculature expansion has been shown to precede the expansion of the epithelium following castration and androgen treatment, suggesting the importance of endothelial derived signals or epithelial growth [15]. We have recently shown that microvessels are in close proximity with TDLUs [16]. A detailed description of the epithelial-endothelial interactions in the human breast gland however, has until recently been largely neglected. There are, however, few reports describing *in vitro *the interaction between endothelial- and epithelial cells in the human breast. Shekhar *et al. *[17,18] showed that interaction between endothelial cells and premalignant breast epithelial cells was necessary to allow sufficient proliferation of endothelial cells as well as to induce branching ductal-alveolar morphogenesis and hyperplasia of premalignant cells [17,18]. In these studies, they used human umbilical vein endothelial cells (HUVEC) instead of organ-specific endothelial cells. It is becoming increasingly recognized that endothelial cells from different organs vary in terms of morphology, marker expression and metabolic properties [19-23] highlighting the need to use organotypic endothelial cells in co-cultures with breast epithelial cells. We have recently improved the isolation protocol and the culture conditions for long term culture of breast endothelial cells (BRENCs) [16]. In this study, we describe a novel three dimensional co-culture system, where primary breast endothelial cells are seeded together with epithelial cells in three dimensional laminin rich gel. We provide evidence that BRENCs can induce proliferation of breast epithelial cells in three-dimensional culture. Furthermore, in co-culture with endothelial cells a subpopulation of luminal epithelial cells form bigger acini/ducts with larger lumens. Seeding normal and cancerous epithelial cells in rBM at clonal dilution with endothelial cells resulted in increased cloning efficacy and larger colony size. This data suggests that endothelial cells in addition to providing nutrient and oxygen to tissues, might be an important microenvironmental factor for normal morphogenesis and cancerous growth in the human breast gland.

## Methods

### Establishment of primary cell culture

Breast tissue specimens were obtained from reduction mammoplasties with informed consent from patients and approval from the National Bioethics Committee of Iceland, Reference number VSNa2001050056. Primary epithelial cells were processed as previously described and cultured on collagen I (Inamed, Gauting, Germany) coated culture flasks (BD Biosciences, Bedford MA) in serum free chemically defined medium (CDM3) [24,25]. Primary breast endothelial cells were isolated from the organoid supernatant as previously described [16]. Briefly, following centrifugation at 1000 rpm for 5 minutes, capillary organoids were isolated using CD31 coated magnetic beads (Invitrogen). Primary endothelial cells were cultured on collagen coated flasks in EGM-2 medium (Lonza, Basel, Switzerland) supplemented with 30% FBS (Invitrogen), heparin, FGF-2, EGF- VEGF, IGFR3, ascorbic acid and hydrocortisone. FBS concentration was reduced to 5% after 2 passages, this medium will be referred to as EGM5.

### Isolation of luminal- and myoepithelial cells

Luminal- and myoepithelial cells outgrown from organoids were isolated with MACS cell sorting system (Miltenyi Biotech, Bergisch Gladbach, Germany), with specific mAb for each cell type (see table 1). EpCAM and MUC-1 were used to isolate luminal epithelial cells and Thy-1 and β 4 integrin for myoepithelial cells. Purified luminal- and myoepithelial cells were cultured on CDM3 and -4 respectively as previously described [24].

**Table 1:** list of antibodies usied in the study

Antibody	Clone	Species	Isotype	Company
**β4-integrin**	3E1	Mouse	IgG1	Millipore
**CD10**	SS 2/36	Mouse	IgG1	Dako

**CD31**	JC/70A	Mouse	IgG1	Dako
**ck14**	LL002	Mouse	IgG3	Abcam

**ck19**	A53-B/A2	Mouse	IgG2a	Abcam
**cl-caspase-3**	Polyclonal	Rabbit	IgG	Cell Signalling

**EpCAM**	VU1D9	Mouse	IgG1	Novocastra
**ki67**	Polyclonal	Rabbit	IgG	Abcam

**MUC-1**	115D8	Mouse	IgG2b	Biogenesis
**thy-1**	ASO02	Mouse	IgG1	Dianova
**ZO-1**	1A12	Mouse	IgG1	Zymed

### Cell lines

Human breast cell lines MCF10A, D382 [26], MCF7, T47-D and MDA-MB-231 were used in three dimensional culture (see table 2 for details). MCF10a and D382 were maintained on H14 medium [27]. MDA-MB-231, T47-D and MCF-7 were maintained on ATCC recommended culture medium.

**Table 2:** List of cell lines used in the study

	Cell line	Origin	Phenotype
**Normal like cell lines**	MCF10a	F	Basal/Mixed
	
	D382	RM	Luminal

**Cancer cell lines**	MCF7	IDC (PE)	ER+
	
	T47-D	IDC (PE)	ER+
	
	MDA-MB-231	AC (PE)	ER- MES

**F**: Fibrocystic disease, **RM**: Reduction mammoplasty, **IDC**: Invasive ductal carcinoma, **PE**: Pleural effusion, **AC**: Adenocarcinoma, **ER**: Estrogen Receptor, **MES**: Mesenchymal			

### Three-dimensional cell culture

1 × 10^4 ^primary epithelial cells were suspended in 300 μl rBM along with 2 × 10^5 ^endothelial cells and seeded in a 24-well plate. After incubation at 37°C for 30 minutes the cultures were supplemented with EGM5 medium. Co-cultures were maintained for 14 days and culture medium was changed three times per week.

The epithelial cell lines MCF10A, D382, MCF7, T47-D and MDA-MB-231 (table 2) were seeded at a clonal density (500 cells per gel) with 2 × 10^5 ^BRENCs and cultured as described above. Colony size and number was measured on days 5, 9 and 13.

To determine dose effect of endothelial cells in co-culture, BRENCs were seeded at increasing concentrations - ranging from 1,000 cells to 200,000 cells - with 250 MCF10A cells. Colony size and number was measured on day 10.

To prevent direct cell-cell contact, BRENCs were seeded on a 0.4 μm pore size Transwell (TW) filter (Corning Life Sciences, Lowell, MA) and cultured in a 12 well plate for 3 days. Epithelial cells (500 cells per well) were then seeded into 100 μl rBM in a separate plate and placed in an incubator at 37°C for 10 minutes. Confluent BRENCs on TW filters were then transferred on top of the gels. Cultures were maintained on EGM5 medium for 8 days.

Gradient co-cultures were conducted using 7 × 10^4 ^BRENCs embedded into 100 μl of rBM and seeded in a 4-well chamber slide. 3 × 10^3 ^epithelial cells were seeded in separate 100 μl rBM and placed in the same well as the BRENCs, allowing the gels to merge in the centre, achieving a gradient in the densities of the two cell types. The chamber slide was then placed in an incubator at 37°C for 20 minutes and supplemented with 1 ml EGM5. Cultures were maintained for 10 days.

### Immunochemistry

Gels were frozen in n-hexane at the end of the culture period. For cryosectioning, gels were mounted in O.C.T. medium and sectioned in 9 μm slices in a cryostat. Primary tissue samples were sectioned in 9 μm slices for immunofluorescence and 5 μm slices for DAB staining. Cryostat sections were fixed in methanol at -20°C for 10 minutes and incubated with primary antibodies (table 1) mixed in PBS+10% FBS for 30 minutes. Slides were incubated with isotype specific fluorescent antibodies (Alexa fluor (AF, 488 (green), 546 (red) Invitrogen) mixed in PBS+10% FBS for 30 minutes in the dark. The specimens were then incubated with a fluorescent nuclear counterstain (TO-PRO-3, Invitrogen) and mounted with coverslips using Fluoromount-G (Southern Biotech). Co-culture gels were stained in a similar manner, with an initial blocking step using IF blocking solution [28] (10% goat serum (Invitrogen) and 1% Goat anti Mouse F(ab')2 Fragments (Invitrogen) in PBS) for 30 minutes. For F-actin staining sections were fixed in 3.7% formaldehyde for 10 minutes and permeabilized with 0.1% Triton-X-100 in PBS for 5 minutes. Slides were then incubated with AF488 conjugated Phalloidin (Invitrogen) for 30 minutes and counterstained with TOPRO-3.

### In gel staining of endothelial cells

Endothelial cells were seeded on top or into rBM and cultured for two weeks. Visualization of CD31 was performed after 24 hours and Ac-LDL uptake after two weeks. For CD31 visualization, gels were fixed in methanol at -20°C for 10 minutes. Nonspecific binding was blocked using IF blocking solution for 30 minutes, followed by an overnight incubation with anti CD31 antibody. Secondary AF488 IgG1 antibody was incubated for 2 hours, followed by TOPRO-3 counterstaining for 15 minutes. LDL uptake of embedded endothelial cells was visualized by incubation of Alexa Fluor 488 AcLDL conjugate (Invitrogen) for 5 hours. Immunofluorescence was visualized using a Zeiss LSM 5 Pascal laser scanning microscope. See table 1 for list of antibodies used in this study.

### Imaging and statistical analysis

All three-dimensional culture experiments were performed in triplicate for statistical accuracy. Imaging was performed using a Leica DMI3000 microscope and a Leica 310FX imaging system. Populations were compared using an unpaired two-tailed t test. Sample distribution was tested using an F-test. Welch correction was used for t-tests of samples with unequal variation. Graphs were created in Microsoft Excel. Error bars represent the standard error of the mean (SEM) unless stated otherwise.

## Results

### Breast endothelial cells cultured in rBM are quiescent but metabolically active

**Figure 1:**
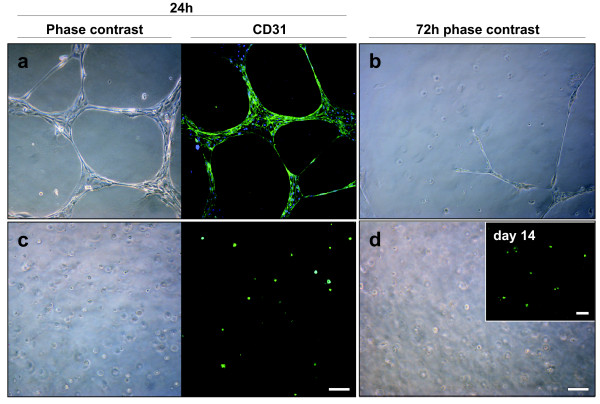
**Endothelial cells stay quiescent but metabolically active when cultured within rBM**. When BRENCs are seeded onto rBM they form network structures within a few hours that disassociate from the rBM within 72 hours (upper panel). In contrast BRENCS that are embedded into rBM (lower panel) stay quiescent but metabolically active for a prolonged time in culture. Fluorescent staining in a and c reveals CD31 expression. Note the insert in (**d**) that demonstrates the uptake of Ac-LDL at day 14. Bars = 50 μm.

When breast endothelial cells (BRENCs) are cultured on top of rBM they form a dense, capillary-like, network shortly after seeding (Figure 1a). However, after approximately 72 hours these structures detach from the gel (Figure 1b). The short lifespan of endothelial cells in this assay limits their use in long-term culture. In contrast, BRENCs that are embedded into the rBM appear as small round viable cells (Figure 1c, left). In these culture conditions the BRENCs stay proliferative quiescent but metabolically active for an extended time period (at least 14 days) (Figure 1d). Immunofluorescence staining demonstrate that BRENCs retain their marker expression in rBM as evidenced by CD31 staining (Figure 1c, right) and stay metabolically active as shown by uptake of acetylated low density lipoprotein (Ac-LDL) after two weeks in culture (Figure 1d insert). Prolonged proliferative quiescence of endothelial cells when seeded within rBM provides an opportunity to analyze endothelial contributions to epithelial growth and morphogenesis.

### BRENCs facilitate growth of primary luminal and myoepithelial cells

**Figure 2:**
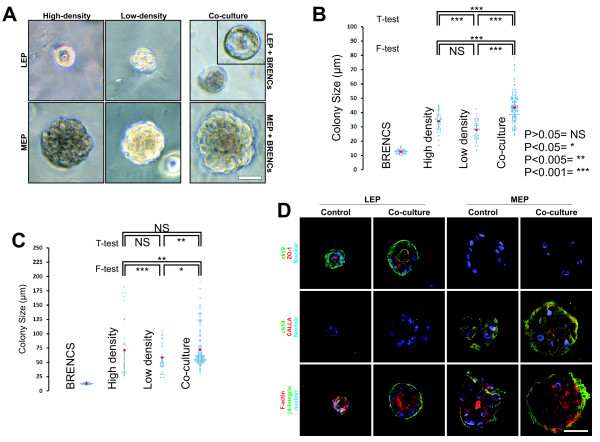
**BRENCs stimulate growth and morphogenesis of primary breast epithelial cells**. **A. BRENCs stimulate growth of both luminal epithelial and myoepithelial cells in rBM**. *Upper panel*: At high density, LEP cells form colonies with a small central lumen. At low density, no lumen formation is seen. In co-culture of low density LEP and BRENCS, large colonies with a large central lumen can be seen. *Lower Panel*: At high density, MEP cells form solid round colonies. At low density, colony size is reduced, but increases in co-culture with BRENCs. Bar = 50 μm. **B. Scatter plot demonstrate that co-culture of BRENCs and LEP result in larger colony size than LEP alone cultured in low and high density**. Colony size in high density control was significantly greater than in low density control. Average colony size in co-culture (n = 135) was significantly increased when comparing with high and low density controls (n = 45, both). Sample variance was also increased significantly when comparing controls and co-culture, but not between the controls. For samples of unequal variance, Welch's correction was used when performing the T-test. **C. Scatter plot show large variance in MEP culture size with and without BRENCs**. Colony size in high density control (n = 45) was significantly higher than in low density control (n = 45). Sample variance was also different. A significant difference was seen when comparing low density control and co-culture (n = 135), but not in high density control and co-culture. **D. Phenotypic characterization of luminal epithelial and myoepithelial colonies with and without BRENCs in rBM**. Immunofluorescence of cryosectioned colonies revealed that LEP colonies express CK19 and the tight junction protein ZO1 whereas MEP colonies were negative. MEP colonies, in contrast are positive for CK14 and CALLA. β4-integrin delineates the basal surface of both LEP and MEP colonies. Note the strong F-Actin staining subapical in co-culture of LEP and BRENCs. Cells were counterstained with TO-PRO-3 (blue). Bar = 50 μm.

BRENCs and isolated primary luminal epithelial (LEP) or myoepithelial cells (MEP) were embedded within rBM and co-cultured for 14 days (Figure 2). When breast epithelial cells were cultured alone in rBM at high density (10^5 ^cells within 300 μl rBM), LEPs formed acini-like colonies with a small central lumen (Figure 2A) as has previously been shown [29], whereas MEPs formed solid round colonies. At lower densities (10^4 ^cells per 300 μl rBM) growth was reduced and limited lumen formation was observed in LEP cultures. In contrast, co-culture of epithelial cells seeded at low density with endothelial cells, resulted in increased colony size, in both LEP and MEP co-cultures compared to low density control (Figure 2A). Interestingly, a dramatic increase in lumen size was observed in a subpopulation of LEP colonies in co-culture, (Figure 2A insert). Scatter plot reveals an increase in colony size in co-culture compared to both high density and low density LEP cultures (Figure 2B). Average colony size in high density and low density monoculture was 34 and 28 μm, respectively (Figure 2B). In contrast, average colony size in co-culture of BRENCs and LEP was 44 μm. In MEP cultures average colony size in high density and low density MEP culture was 71 and 58 μm, respectively (Fig 2C). In contrast, average colony size in co-culture of BRENCs and low density MEP was 72 μm. Interestingly, there was much more variation in colony size within each MEP culture than LEP culture. This data indicate that endothelial cells can signal to both luminal and myoepithelial cells to form larger colonies in co-culture than when cultured alone. Immunophenotypic characterization of high density (HD) culture and co-culture show clear apical to basal polarization in luminal epithelial cells. Luminal colonies are polarized with a central lumen and basally located nuclei, as evidenced by F-actin and nuclear stain (Figure 2D). No lumen formation is observed in MEP cultures. LEP colonies display basal polarization as seen with basal β4-integrin staining of both HD control and co-culture colonies. Apical polarization of LEP colonies is evidenced by staining against ZO-1. MEP colonies are negative for both CK19 and ZO1. Similar staining pattern is seen in HD and co-culture. Myoepithelial colonies also show a basal polarization as judged by β4 integrin expression (Figure 2D) but no apical polarization is observed.

### Clonal colony formation is enhanced by BRENCs in normal and malignant breast epithelial cell lines

**Figure 3:**
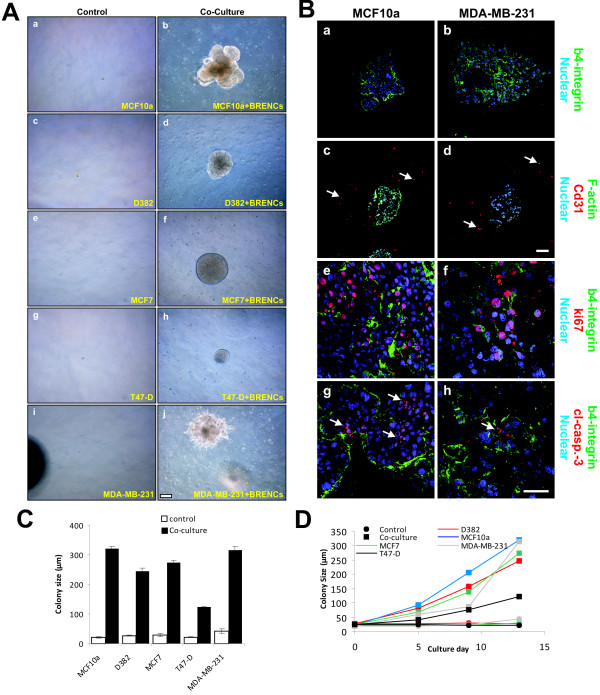
**BRENCs induce clonal growth of normal and malignant breast epithelial cell lines**. **A) BRENCs generate positive environment for clonal growth of breast epithelial cells**. MCF10A, D382, MCF7, T47-D and MDA-MB-231 cells were seeded at a clonal dilution, alone or in co-culture with BRENCs. When cultured alone there was very limited colony formation, most seeded cells stayed as non-proliferative single cells (**a**, **c**, **e, g, i**). In contrast, co-cultures of BRENCs with all cell lines resulted in dramatic increase in colony growth. MCF10A cells formed multiacinar-like structures (**b**), whereas D382 formed solid round colonies (**d**). both MCF7 and T47-D formed solid round colonies (**f**, **h**). MDA-MB-231 formed mesenchymal like colonies (**j**). Bar = 100 μm. **B) Immunophenotypic characterization of endothelial-epithelial co-cultures**. β4-integrin staining reveals the different phenotype of MCF10A and MDA-MB-231, where MCF10A forms dense multiacinar structures delineated by β4-integrin expression but MDA-MB-231 forms colonies of loosely connected cells with diffused staining pattern of β4-integrin. (**a-b**). Note the expression pattern of β4-integrin. CD31 staining shows the distribution of BRENCs around the colonies (**c-d**). ki67 expression levels are similar in both cell lines (**d-e**) and minimal expression of cleaved caspase 3 is seen (**g-h**) Bar = a-d 100 μm and e-h = 50 μm. **C) Colony size is increased in co-cultures with BRENCs**. Colony size (n > 100 for all cell lines) of epithelial cells co-cultured with BRENCs increased significantly compared with controls (P < 0.0001 for all co-cultures). MCF10A gave the biggest response, with a 15.5 fold increase in colony size, averaging at 320.6 μm diameter. Colony size varied greatly between cell lines. **D) Colony growth over time**. Most cell lines followed a linear growth pattern. Note however, the drastic change in growth of MDA-MB-231 cells after day 9 (light grey line).

In order to analyze the effects of endothelial cells on proliferative and morphogenic phenotypes of established cell lines, several normal and cancerous breast epithelial cell lines were tested (table 2). When seeded within rBM at a clonal dilution (500 cells per 300 μl rBM), normal and malignant epithelial cells show limited or no proliferation (Figure 3A, left panel). In contrast, when co-cultured with BRENCs, a significant (P < 0.0001) increase in colony size is observed in all tested cell lines (Figure 3A, right panel). The phenotype of colonies in co-culture with BRENCs differs between cell lines, ranging from multiacinar-like structures seen in MCF-10A, solid round (D382, MCF-7 and T47-D) and mesenchymal-like structures seen in MDA-MB-231 co-cultures (Figure 3A, right panel). When cryosectioned and immunostained against β4 integrin it was possible to see the organized and disorganized cell clusters in MCF-10A and MDA-MB-231, respectively (Figure 3B, a-b). In MCF10a cultures, β4 integrin expression is only seen on the periphery of individual acini, whereas in MDA-MB-231 cultures expression is ubiquitously seen, demonstrating a lack of polarity. Immunostaining for CD31 demonstrated the presence of BRENCs as single cells close to the epithelial colonies (Figure 3B, c-d). Immunostaining against ki67 demonstrates high levels of cell proliferation in both MCF-10A and MDA-MB231 colonies in co-culture (Figure 3B, e-f). The levels of apoptosis are low in both cell lines as evidenced by low staining for cleaved-caspase-3 (Figure 3B, g-h). Figures 3C and 3D show the colony size formed in co-culture between different epithelial cell lines and BRENCs compared to controls. A highly significant (p < 0.0001) increase in colony size was seen under co-culture conditions for all cell lines tested (Figure 3C). Colony size continued to increase throughout the culture period (Figure 3D). MDA-MB-231 colony size was dramatically increased from day 9 indicating possible endothelial independent effects after the colony has reached a certain size threshold.

**Figure 4:**
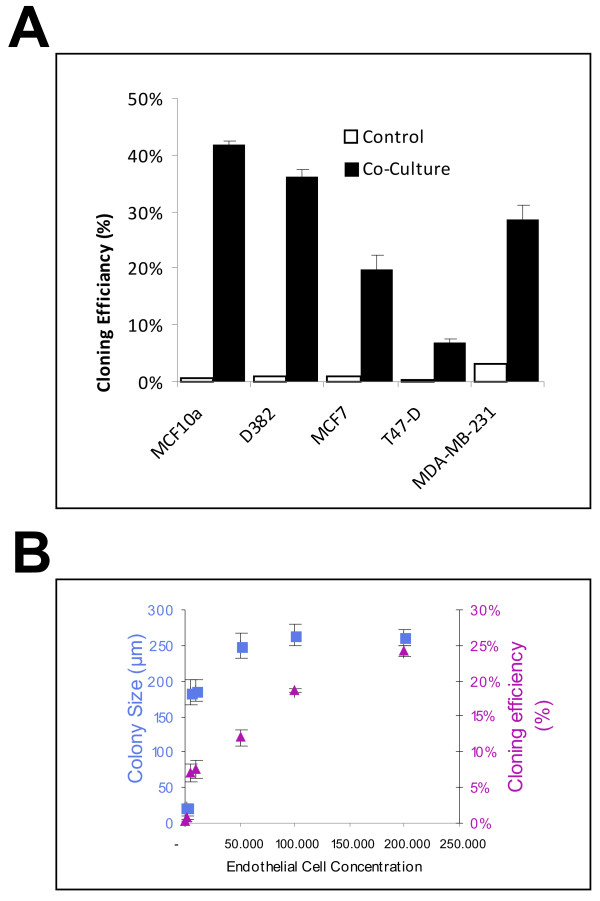
**BRENCs enhance cloning efficiency of normal and malignant breast epithelial cell lines in co-culture**. **A) Colony formation (cloning efficiency) is greatly increased in co-cultures**. MCF10A cells' colony forming ratio increased from 1% to 41.9%. MDA-MB-231 colony formation increased from 3.2% to 28.6%. Overall, increase in colony formation was greater for the normal cell lines than for the cancer cell lines. **B) Density of BRENCs determines the cloning efficiency of MCF10A cells in co-culture**. Increasing the density of BRENCs in co-culture resulted in an increase in colony formation (triangle). Colony size reached an apparent plateau at a BRENCs density of 50,000 (square box).

In epithelial cultures without BRENCs, there was very limited colony formation, whereas in the co-culture conditions there was a marked increase in cloning efficiency. Figure 4 depicts the effects of BRENCs on cloning efficiency in the different cell lines, i.e. the number of colonies relative to the number of cells seeded. The cloning efficiency increased from less than 5% in controls to between 9% and 41% in co-cultures (Figure 4A). To analyze whether proliferative effects were dependent on the amount of BRENCs in co-cultures, MCF10a cells were embedded in rBM with increasing amount of BRENCs. In low density BRENCs co-cultures, there is limited cloning efficiency (less than 5%) and proliferation (colonies <100 μm). When BRENCs' density was increased, cloning efficiency increased in a near-linear fashion, reaching its highest level with 200,000 BRENCs (24%) whereas colony size stopped increasing much earlier, reaching a plateau (~250 μm) at 50,000 BRENCs (Figure 4B).

### Proliferative signals from BRENCs are delivered via soluble factors

**Figure 5:**
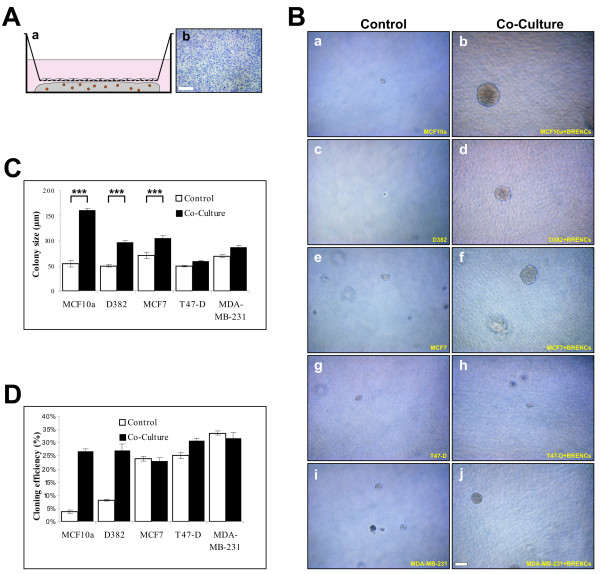
**Endothelial-derived effects are mediated trough soluble factors**. **A) Schematic figure of a Transwell co-culture assay**. BRENCs (grey) were seeded on Transwell (TW) filter inserts and allowed to reach confluency. Epithelial cells (orange) were embedded into Matrigel and seeded in the lower chamber (**a**). Hematoxylin stained BRENCs on a TW insert (**b**). Bar = 200 μm. **B) Phase contrast images of controls and co-cultures in rBM**. BRENCs were able to stimulate proliferation of MCF10a (**a**, **b**) and D382 (**c**, **d**) when separated by a Transwell filter. BRENCs were also able to stimulate proliferation of MCF7 (**e**, **f**), T47-D (**g**, **h**) and MDA-MB-231 (**i**, **j**) when separated by a Transwell filter. Bar = 50 μm. **C) Epithelial colony size is increased in co-culture with BRENCs**. A significant (P < 0.0001) increase in colony size of the cell lines MCF10a, D382 and MCF7 was seen in co-cultures (black bars) compared to controls (white bars). No significant proliferative effect was seen in T47-D and MDA-MB-231 co-culture with BRENCs. **D) Cloning efficiency of normal breast epithelial cells is enhanced in co-culture with BRENCs**. Colony formation was increased for the non-malignant cell lines only (MCF10a and D382). In contrast, similar colony count was seen in controls and co-cultures for the cancer lines.

To discriminate between direct contact and soluble factors in co-culture we used Transwell (TW) filters and a gradient co-culture system to physically separate the two cell populations. Endothelial cells were plated in monolayer on TW filters and allowed to grow to confluency. 500 epithelial cells were then seeded into 100 μl rBM. Endothelial coated TW filters were next placed on top of the gel (Figure 5A). Some colony formation was also evident in control cultures in this setup and could be explained by better diffusion of growth factors into the gel from the culture medium due to a higher area/volume. Colony size was significantly larger in Transwell separated co-cultures of the normal cell lines MCF10A and D382, as well as MCF-7 (P < 0.0001) compared to co-cultures of the other malignant cell lines T47-D and MDA-MB-231, where no significant growth increase was detected.(Figure 5B and 5C). Cloning efficiency was increased in co-cultures with the normal epithelial cell lines MCF10a and D382, but not with the malignant epithelial cell lines. This possibly demonstrates a lower need for growth stimulation of the cancerous cell lines in comparison to the normal cell lines (Figure 5D).

**Figure 6:**
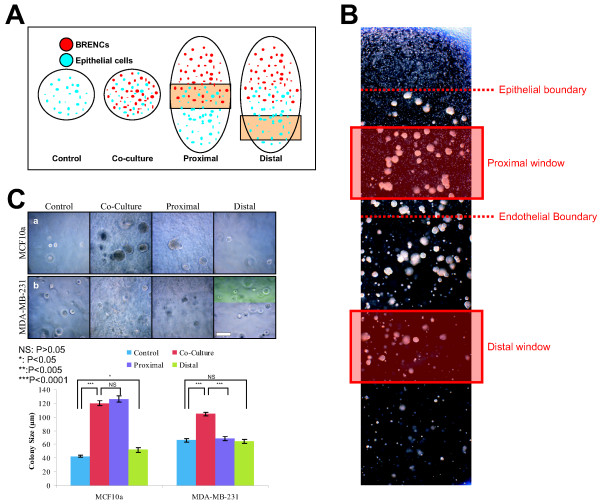
**Proliferation effects decrease in relation with distance from endothelial cells**. **A) Schematic representation of BRENCs and epithelial cells in gradient co-culture**. BRENCs and epithelial cells were embedded into separate gels on culture plates. The gels were allowed to fuse before culture medium was added. **B) Composite image of the gradient co-culture demonstrating the epithelial and endothelial boundaries**. Phase contrast image demonstrates the spatial distribution of epithelial colonies. The size of the colonies decreases proportionally with the distance from BRENCs. The distal colony growth, however, demonstrates that the endothelial effects are mediated at least partially by soluble factors. **C) Effects of endothelial cells are most prominent at close distance to epithelial cells**. Gradient co-culture show prominent proximal effects in MCF10A. The effects are however still present distally. No distal effects were seen in co-culture of BRENCs and MB-MDA-231. The apparent difference between co-culture and proximal windows can possibly be explained by the earlier appearance of mesenchymal colonies in co-culture.

To examine the spatial extent of BRENCs growth signals we setup a co-culture assay (Figure 6A) with a gradient in the densities of both BRENCs and epithelial cells. MCF10a co-culture showed that colony growth was most prominent in close contact with BRENCs and was comparable to regular co-culture but distal effects, however, were also visible (Figure 6B and 6C). This further demonstrated that BRENCs mediate the proliferative effects through soluble factors but the effects diffuse slowly through the gel. Growth of MDA-MB-231 showed a different pattern, where no significant proliferative effect was seen in either proximal or distal windows (Figure 6B and 6C). The appearance of spindle shaped colonies in the proximal window was also rarer than in co-culture, perhaps explaining this apparent size difference.

## Discussion

In this paper we have presented a novel three dimensional co-culture system that can be used to analyze cell-cell interaction in heterotypic co-culture. We have demonstrated that isolated primary breast endothelial cells exert a density dependant proliferative effect on epithelial cells when co-cultured. These growth signals are conveyed by soluble factors that disperse from the endothelial cells.

Paracrine interactions are important in the stromal-epithelial crosstalk within the breast gland. Various stromal cells such as fibroblasts produce growth factors and extracellular matrix that influence breast morphogenesis and cancer progression but very little is known about the inductive signals from vascular endothelium. Our data supports the notion that stroma is a vital regulator of tissue morphogenesis and could have a role in cancer progression in the human breast and thus adds a new key player, endothelial cells to this scenario. Studies on epithelial-endothelial interactions in the human breast are lacking. In contrast, studies in mice have shown that angiogenesis precedes the growth of epithelium during puberty and pregnancy when mammary epithelium undergoes a dramatic growth phase [30]. This suggests that endothelium may contribute to the breast morphogenesis. During pregnancy the mammary epithelium and its supporting intra-lobular vasculature rapidly expands to prepare for lactation, resulting in dramatic changes in the microenvironment [31]. The vasculature of the lactating gland is composed of well-developed capillary meshwork enveloping the secretory acini with basket-like structures [30]. During involution, apoptotic cell death returns the breast gland from active to resting state [30]. These morphological changes are also seen during each menstruation cycle where the breast gland undergoes a miniature version of this cycle observed during pregnancy, lactation and involution [32]. Vascular networks exist in most tissues where endothelial cell are in prime position to interact with parenchymal cells such as the epithelial cells. Indeed, recent data from various organs such as liver, pancreas, brain and bone marrow indicate that organ specific endothelial cells are important for fate control of stem cells, organogenesis and tissue maintenance (reviewed in [33]). Lammert *et al. *showed that endothelial cells are important for both pancreas and liver development before the endothelium takes up its usual roles [14]. In the nervous system Shen *et al. *[13] demonstrated that endothelial cells were enriched in the niche occupied by neural stem cells and that these endothelial cells regulate nerve stem cell proliferation and induce these stem cells to become neurons *in vitro*. Lai *et al. *[34] showed that endothelial cells induced proliferation and functional differentiation in embryonic stem cell-derived neural progenitor cells. In the bone marrow, hematopoietic stem cells are regulated by the vascular niche [35]. *In vitro *experiments have shown that endothelial cells can provide the right environment for growth and differentiation of megakaryocytes [36].

In our 3D culture model BRENCS remain proliferatively quiescent but metabolically active and generate a stimulatory microenvironment for epithelial cells. This quiescence enables visualization of proliferating cells over a long time period, as the endothelial cells themselves do not form colonies that would limit visibility in the assay. Improvement of our *in vitro *three-dimensional cell culture model, for example incorporating fibroblasts is important. Nonetheless, these models remain superior systems to approach the situation found *in vivo*. Animal models, in particular mice, have provided extensive information regarding mammary development and cancer progression. Human and mouse mammary glands, however, have distinct differences [2]. In addition, an inherent limitation to *in vivo *models is the lack of information regarding cell-cell and cell-stroma interactions. Monolayer cultures (2D), due to their lack of physiological context are not suitable to study tissue morphogenesis. Breast epithelial cells cultured in 2D fail to form acinar-like structures and lose tissue specific differentiation such as apical-basal differentiation. In contrast, 3D models have proven to be highly relevant when studying the tissue morphogenesis and cancer progression where they add critical elements not found in conventional two dimensional cell culture systems [37].

The observation that BRENCs stimulate a subpopulation of primary luminal epithelial cells to form colonies with a larger lumen is of interest and could indicate that these epithelial cells were derived from a ductal part of the epithelium rather than the small lobuli-derived acini. Using a Transwell assay we demonstrated that the proliferative effects of BRENCs are delivered by soluble factors. However, these factors do not diffuse effectively through the gel, and are most prominent at close proximity. These factors remain to be identified. Recent studies on endothelial-epithelial interaction by Neiva *et al. *have identified factors produced by endothelial cells that enhance migration and survival of epithelial cells [38]. The appearance of spindle shaped MDA-MB-231 colonies occurred most often in co-culture with complete mixing of the cell types (Figure 4), whereas in both the Transwell and gradient co-cultures the appearance rates were much lower (not shown). This suggests that even though proliferative effects are conferred, they are not as strong as in close cell-cell contact.

## Conclusions

Our co-culture model may help define some of the key components involved in heterotypic cell-cell interactions in normal breast morphogenesis and cancer progression. This model might be relevant for hard to culture cell types such as primary breast cancer cells where one might be able to grow these cells more readily *in vitro*. This study strengthens the notion that to understand tissue maintenance and tumor progression it is important to gain information on stromal components interacting with the epithelial cells. It is clear from other tissues that endothelial cells play an important role in organogenesis and tissue maintenance. Our data provides important hints that this might also be true in the breast gland. Furthermore, endothelial cells and their interaction with malignant breast cells might be an important factor to take into consideration in breast cancer biology.

## List of abbreviations

BRENC: Breast endothelial cell; LEP: Luminal epithelial cell; MEP: Myoepithelial cell; 3D: Three dimensional; rBM: Reconstituted basement membrane; TDLU: Terminal duct lobular unit; ECM: Extracellular matrix; CDM: Chemically defined medium; TW: Transwell; PBS: Phosphate buffered saline.

## Competing interests

The authors declare that they have no competing interests.

## Authors' contributions

SI planned and performed the experiments, performed data interpretation and statistical analysis and participated in writing the paper. VS, AJRF and MKM planned and discussed the experiments and participated in data interpretation and writing the paper. JGJ and JK provided access to primary breast tissue from reduction mammoplasties. TG planned and coordinated the study, participated in data interpretation and wrote the paper. All authors read and approved the final manuscript.
